# Reproducible Synthesis of Biocompatible Albumin Nanoparticles Designed for Intra-articular Administration of Celecoxib to Treat Osteoarthritis

**DOI:** 10.1021/acsami.4c02243

**Published:** 2024-03-14

**Authors:** Rumi Khandelia, Tom Hodgkinson, Daniel Crean, Dermot F. Brougham, Dimitri Scholz, Hossam Ibrahim, Susan J. Quinn, Brian J. Rodriguez, Oran D. Kennedy, John M. O’Byrne, David J. Brayden

**Affiliations:** † UCD School of Veterinary Medicine, University College Dublin, Belfield, Dublin D04 V1W8, Ireland; ‡ UCD Conway Institute, University College Dublin, Belfield, Dublin D04 V1W8, Ireland; § Department of Anatomy and Regenerative Medicine, Royal College of Surgeons in Ireland, 123 St. Stephen’s Green, Dublin D02 YN77, Ireland; ∥ UCD School of Chemistry, University College Dublin, Belfield, Dublin D04 V1W8, Ireland; ⊥ UCD School of Physics, University College Dublin, Belfield, Dublin D04 V1W8, Ireland; # National Orthopaedics Hospital Cappagh, Dublin D11 EV29, Ireland

**Keywords:** albumin nanoparticles, celecoxib, drug delivery systems, osteoarthritis management, intra-articular delivery

## Abstract

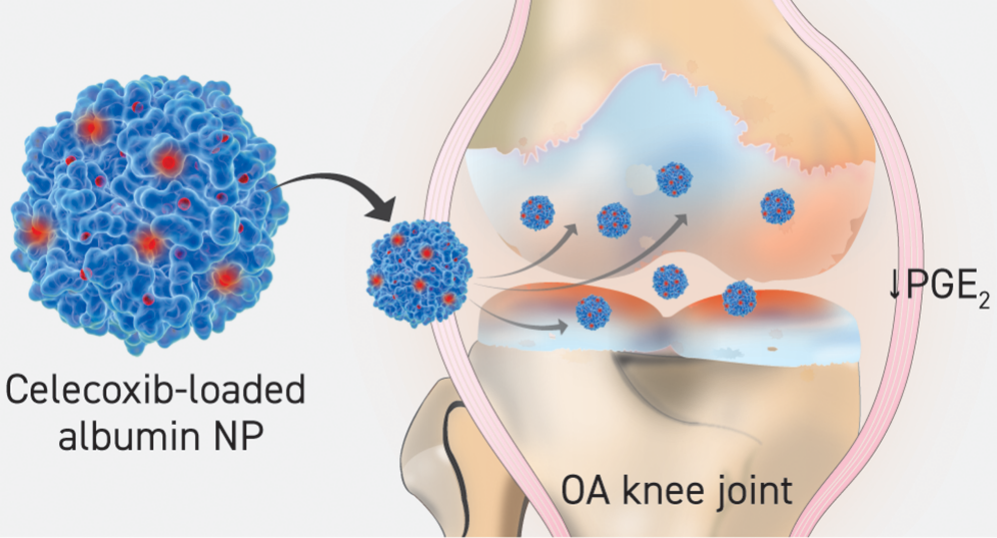

Osteoarthritis (OA) is the most common form of arthritis, with intra-articular (IA) delivery of therapeutics being the current best option to treat pain and inflammation. However, IA delivery is challenging due to the rapid clearance of therapeutics from the joint and the need for repeated injections. Thus, there is a need for long-acting delivery systems that increase the drug retention time in joints with the capacity to penetrate OA cartilage. As pharmaceutical utility also demands that this is achieved using biocompatible materials that provide colloidal stability, our aim was to develop a nanoparticle (NP) delivery system loaded with the COX-2 inhibitor celecoxib that can meet these criteria. We devised a reproducible and economical method to synthesize the colloidally stable albumin NPs loaded with celecoxib without the use of any of the following conditions: high temperatures at which albumin denaturation occurs, polymer coatings, oils, Class 1/2 solvents, and chemical protein cross-linkers. The spherical NP suspensions were biocompatible, monodisperse with average diameters of 72 nm (ideal for OA cartilage penetration), and they were stable over 6 months at 4 °C. Moreover, the NPs loaded celecoxib at higher levels than those required for the therapeutic response in arthritic joints. For these reasons, they are the first of their kind. Labeled NPs were internalized by primary human articular chondrocytes cultured from the knee joints of OA patients. The NPs reduced the concentration of inflammatory mediator prostaglandin E_2_ released by the primaries, an indication of retained bioactivity following NP synthesis. Similar results were observed in lipopolysaccharide-stimulated human THP-1 monocytes. The IA administration of these NPs is expected to avoid side-effects associated with oral administration of celecoxib and to maintain a high local concentration in the knee joint over a sustained period. They are now ready for evaluation by IA administration in animal models of OA.

## Introduction

Osteoarthritis (OA) is the leading cause of chronic disability worldwide.^1^ It can affect any joint of the body, but mainly the hands, knees, hips, and spine joints.^1^ More than 528 million people are affected by OA worldwide.^2^ The average annual cost per patient for OA treatment in US and EU can be up to $12000^1^ in a global market estimated at almost $7 billion.^3^ To date, no treatments have been discovered that can cure or reverse OA.^1^ However, to treat symptomatic pain and inflammation associated with knee OA, analgesics, non-steroidal anti-inflammatory drugs (NSAIDs), hyaluronic acid, corticosteroids, and occasionally, opioids are administered via oral, topical, and intra-articular (IA) routes.^1^ NSAIDs are the most widely used drug class^4^ and are strongly recommended by American Academy of Orthopaedic Surgeons for OA treatment.^5^

There are two types of NSAIDs available to treat OA, non-selective cyclooxygenase (COX) inhibitors and selective COX-2 inhibitors.^4^ Both classes can potentially cause cardiovascular side effects including myocardial infarction, but generally COX-2 inhibitors carry greater risk.^4^ COX-2 inhibitors on the other hand cause less side-effects in the gastrointestinal (GI) tract compared to non-selective inhibitors.^6^ Due to an increased risk of heart attack and stroke, COX-2 inhibitors were taken off the US market almost 18 years ago with the exception of celecoxib (Cel).^7^ This exception was granted because the cardiovascular risk of Cel at the lowest approved oral dose of 100 mg twice daily in capsules was comparable to that of the non-selective COX inhibitors, ibuprofen, dosed up to 800 mg three times daily or naproxen dosed up to 500 mg twice daily.^8^ Cel is also still marketed in EU for OA.^9^ It has a black box warning relating to risks of cardiovascular events and GI bleeding,^10^ even though advantages in reducing the latter were the original basis for its development. Even with these risks, the global market for Cel capsules is expected to reach $82 million by 2028.^11^

One possible way to circumvent the potential cardiovascular and GI side-effects of Cel is to inject it directly into OA knee joints by IA injection.^1,2,12−15^ However, due to its low molecular weight, free Cel is rapidly eliminated from joints within a few hours. As a result, repeated administration of Cel is required to reach therapeutic local concentration, which in turn increases the risk of side-effects.^1,2,12−15^ Moreover, Cel is highly lipophilic and is insoluble in aqueous media, and the free form may lead to crystal formation in the IA compartment, risking crystal deposition and synovitis.^1,14^ One option to counter these problems is to load Cel in a nanoparticulate drug delivery system (DDS) and administer it directly into the OA joint through IA injection.^1,2,12−15^ Several DDSs loaded with anti-inflammatory therapeutic agents including Cel have been explored for potential IA administration for OA treatment with an aim of improving retention time in joints.^1,2,12−20^ The DDSs developed were based on synthetic polymers, polysaccharides, proteins, peptides and lipids.^1,2,12−20^ Corticosteroid-loaded liposomes (Lipotalon)^1^ and poly(lactic-*co*-glycolic acid) (PLG) microspheres (ZILRETTA)^2,12^ have been marketed for knee OA so the principle of IA-administered therapeutics has been established. Regarding limitations, liposome-based DDSs have limited capacity to load lipophilic drugs and are less stable than other DDSs.^1^ Additionally, Lipotalon has not been marketed outside Germany.^1^ For the PLG system, repeated IA administration of ZILRETTA to the knee is not yet FDA-approved^21^ and, as PLG does not bind specifically to a receptor to promote targeting to chondrocytes and synoviocytes, the capacity of PLG microspheres to be retained in the joint may also be limited.^22^

Among the matrices used for developing the DDSs, proteins are advantageous as they contain many functional groups,^23^ and the overall charge of the protein can be tuned by changing the pH of the medium.^24^ Thus, the same protein can interact with multiple hydrophilic and lipophilic molecules via H-bonding, hydrophobic interactions, and electrostatic interactions.^24−26^ Albumin is especially promising as a carrier as it is an integral part of human synovial fluid and is non-immunogenic, biodegradable, and biocompatible.^25,27^ It is also an excellent lyophilized protectant for solid forms of DDSs and allows immediate redispersion of dried DDSs in injectable solutions.^28^ This advantage makes albumin-based DDSs a shelf-stable system, thereby avoiding premature drug release and precipitate formation, occurrences which can impede translation.^28^ Importantly, albumin is also a component of existing pharmaceutical therapeutic products.^28,29^

Researchers have demonstrated the potential use of albumin microparticles and nanoparticles (NPs) as DDS for arthritis treatment, where drug-loaded albumin particles were retained in arthritic joints for a longer period compared to free drugs.^30−36^ This is likely due to the fact that albumin has high affinity for secreted protein acidic and rich in cysteine (SPARC), which is overexpressed in arthritic joints.^30,31,37^ Albumin particles have also been developed for other applications.^38−43^ However, all of the synthesis methods used have some disadvantages. These include use of (a) chemical agents that induce protein cross-linking (e.g., glutaraldehyde),^29−31,38,39^ (b) polymer coatings,^40^ (c) oils or Class 1/2 solvents (as in Abraxane synthesis),^32−36,44^ or (d) temperatures at which denaturation of albumin becomes a factor (≥56 °C).^34,41,42^ Chemical agents that induce protein cross-linking can be toxic^33,45^ or are not present in the FDA Inactive Ingredient/Generally Regarded as Safe Substances Databases, or they may negatively interact with protein and/or drug.^46,47^ Furthermore, coating albumin particles with a polymer may interfere with the drug release profile,^48^ while use of an oil such as liquid paraffin makes synthesis technically difficult as washing with an organic solvent is required.^32,33^ According to International Council for Harmonisation (ICH) guidelines, Class 1 solvents should not be used in pharmaceutical products and if their use is unavoidable, their levels should be restricted at a very low parts per million (ppm) values.^49^ Similarly, Class 2 solvents should only be used in a low ppm range.^49^ Thus, the process controls for maintaining the recommended ppm levels for Class 1 and 2 solvents will be significantly more stringent than Class 3 or 4. Finally, temperatures up to 150 °C are typically used to stabilize albumin particles;^24,34,41,42^ however, albumin starts denaturing at 56 °C,^50^ and drugs such as ibuprofen begins to degrade at 68 °C.^51^ Thus, temperatures lower than 56 °C are preferable for the synthesis of a wide variety of drug-loaded albumin particles. There are two reports where 50 °C was used to stabilize albumin particles.^24,42^ In the first, 48 h was required to stabilize the particles,^42^ which is not economical. Moreover, ethanol was used as a desolvation solvent,^42^ which denatures albumin even more than acetone.^52^ In the second report,^24^ albumin-stabilized gold nanoclusters were used instead of simple albumin to make the NPs, and these nanoclusters have not been FDA-approved. There is only one method, which does not involve any of the above conditions/materials and that is self-assembly.^30,39^ However, particles made by this process were reported to have poor colloidal stability, spontaneously dissociating on dilution.^39^ Thus, there is a need to develop an improved method that can reproducibly synthesize stable albumin NPs in an appropriate size range and without the above-listed disadvantages.

To maximize the efficacy of DDSs for OA treatment, they should be capable of penetrating the dense knee articular cartilage extracellular matrix (ECM), and for this, particle size plays a significant role. The main component of cartilage ECM is collagen II with a network mesh size of 50–100 nm in healthy tissue, which acts as a filter for transport of materials.^13,53^ As OA progresses, this mesh size increases, and DDSs up to 200 nm can penetrate OA cartilage.^53^ On the other hand, it is also reported that any DDS greater than 200 nm possesses a risk of activating the immune complement system.^54^ It has been demonstrated that reducing DDS particle size will improve depth of penetration in cartilage.^13^ Hence, the interfacial challenge is to minimize size while ensuring colloidal stability and also providing sufficient drug loading. Yan et al. observed that albumin-coated peptide-siRNA NPs with diameter in the low nanometer range were able to deeply penetrate the human OA cartilage up to a depth of at least 700 μm, persisting in chondrocyte lacunae for at least 2 weeks.^55^ It is desirable to synthesize Cel-loaded albumin NPs having an average size <200 nm but which are as small as possible to facilitate (i) deep penetration and (ii) penetration into the cartilage in early stages of OA, but with no particles >200 nm in order to avoid inflammatory responses.

**Scheme 1:**
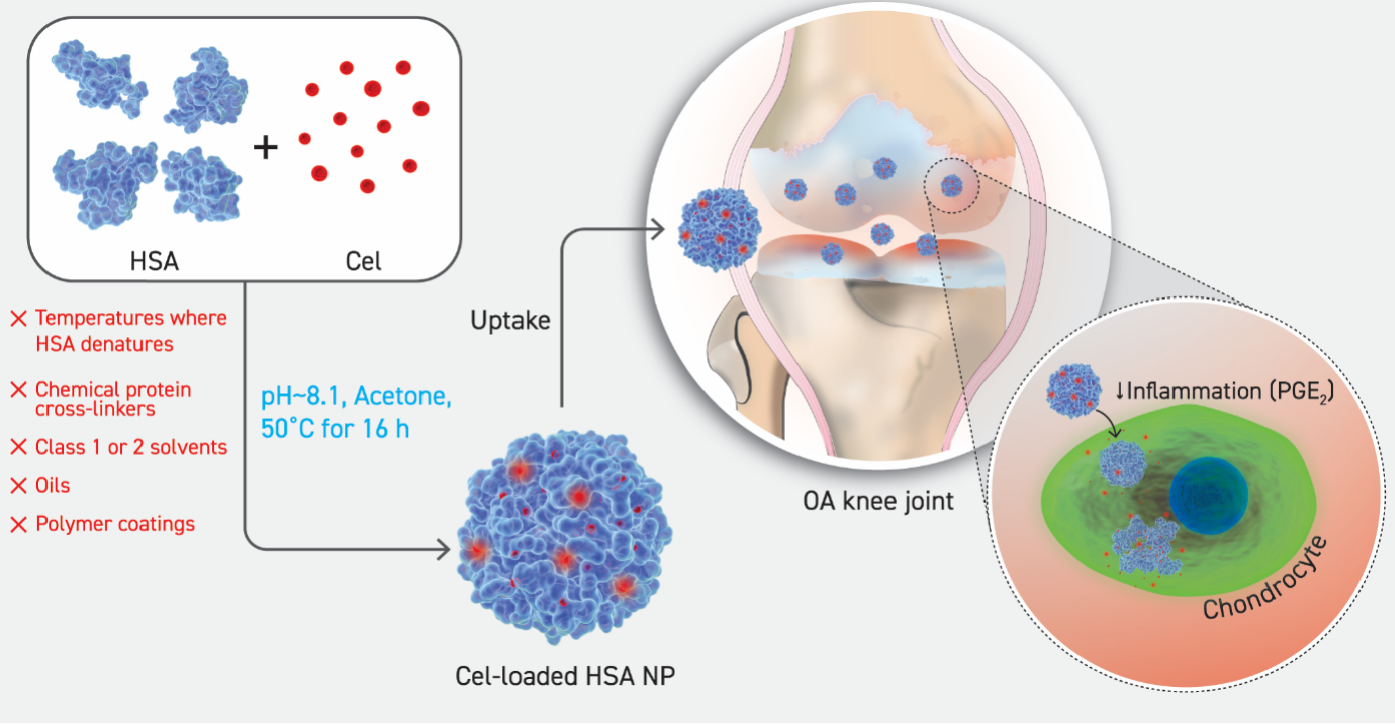
A Schematic Illustration of the Formation of Cel-Loaded HSA NPs, Followed by Uptake by the OA Knee Joint Chondrocytes, Thereby Reducing Levels of PGE_2_ Released by the Cells

Herein, we report the development of a reproducible and economical method to synthesize the colloidally stable Cel-loaded albumin NPs, without the use of chemical agents that induce protein cross-linking, non-approved materials, polymer coatings, oils, Class 1/2 solvents, and temperatures at which denaturation of albumin becomes a factor (≥56 °C). The method yielded spherical, non-agglomerated, monodisperse, biocompatible, and bioactive Cel-loaded human serum albumin (HSA) NPs in the desired size range for OA cartilage penetration and with the demonstrated capacity to be internalized by human articular chondrocytes from the knee joints of OA patients (hOAACs) and to inhibit local COX-2 activity (Scheme 1). The HSA NPs loaded Cel at a higher concentration than that is required to show the therapeutic response in the arthritic joint. These properties of the Cel-loaded HSA NPs along with the ability to be retained in the inflamed joint for a longer time make them an efficient DDS suitable for IA delivery to treat OA.

## Results and Discussion

### Synthesis and Characterization of Cel-Loaded HSA NPs

Cel-loaded HSA NPs were synthesized by varying five factors: HSA concentration, HSA purity, stabilization temperature, incubation time at 50 °C, and pH (Table S1). Different conditions yielded different TEM sizes (*d*_TEM_) of Cel-loaded HSA NPs (Table S1). Multiple sets of reactions were performed to identify optimum conditions yielding the smallest *d*_TEM_, with all the NPs <200 nm and forming stable suspensions. A key aspect proved to be incubation time at 50 °C for stabilization, which showed a trend in reducing size over time.

The optimized Cel-loaded HSA NPs were synthesized using 1% essentially fatty acid free HSA at pH ∼ 8.1, by desolvation using acetone at 30 °C, followed by stabilization at 50 °C for 16 h. The method avoids temperatures at which denaturation of albumin becomes a factor (≥56 °C) and, as noted, is free from polymer coatings, oils, Class 1/2 solvents, and chemical protein cross-linkers. Moreover, our method is economical because acetone, a Class 3 solvent, is allowed to be used in higher concentrations as compared to Class 1/2 solvents in pharmaceutical products,^49^ making the process control easier. In addition, a moderate temperature (50 °C) for 16 h was required to stabilize the NPs. Due to the facile NP synthesis conditions (50 °C, 16 h, use of acetone), the risk of denaturation of HSA in the NPs is reduced.^50,52,56^ Finally, all the materials used in this synthesis are approved by the FDA and EMA. The reproducibility of the synthesis method was checked by synthesizing three different formulations of NPs (batches A1, A2, and A3) using the same batch of HSA (SLBM7779V) and then synthesizing two additional formulations of NPs (batches B1 and C1) using two other batches of HSA (SLCB2530 and SLCF6784). To the best of our knowledge, reproducibility to this level has not been reported before for albumin NPs.

The interaction of Cel with HSA was studied by using fluorescence spectroscopy. The emission spectrum of Cel-loaded HSA NPs recorded upon 242 nm excitation showed a red shift (from 329 to 335 nm) compared to the spectrum of unloaded control HSA NPs (Figure S1), indicating that Cel interacted with HSA in the NPs. Cel is known to interact with HSA primarily through H-bonding.^26^

**Figure 1:**
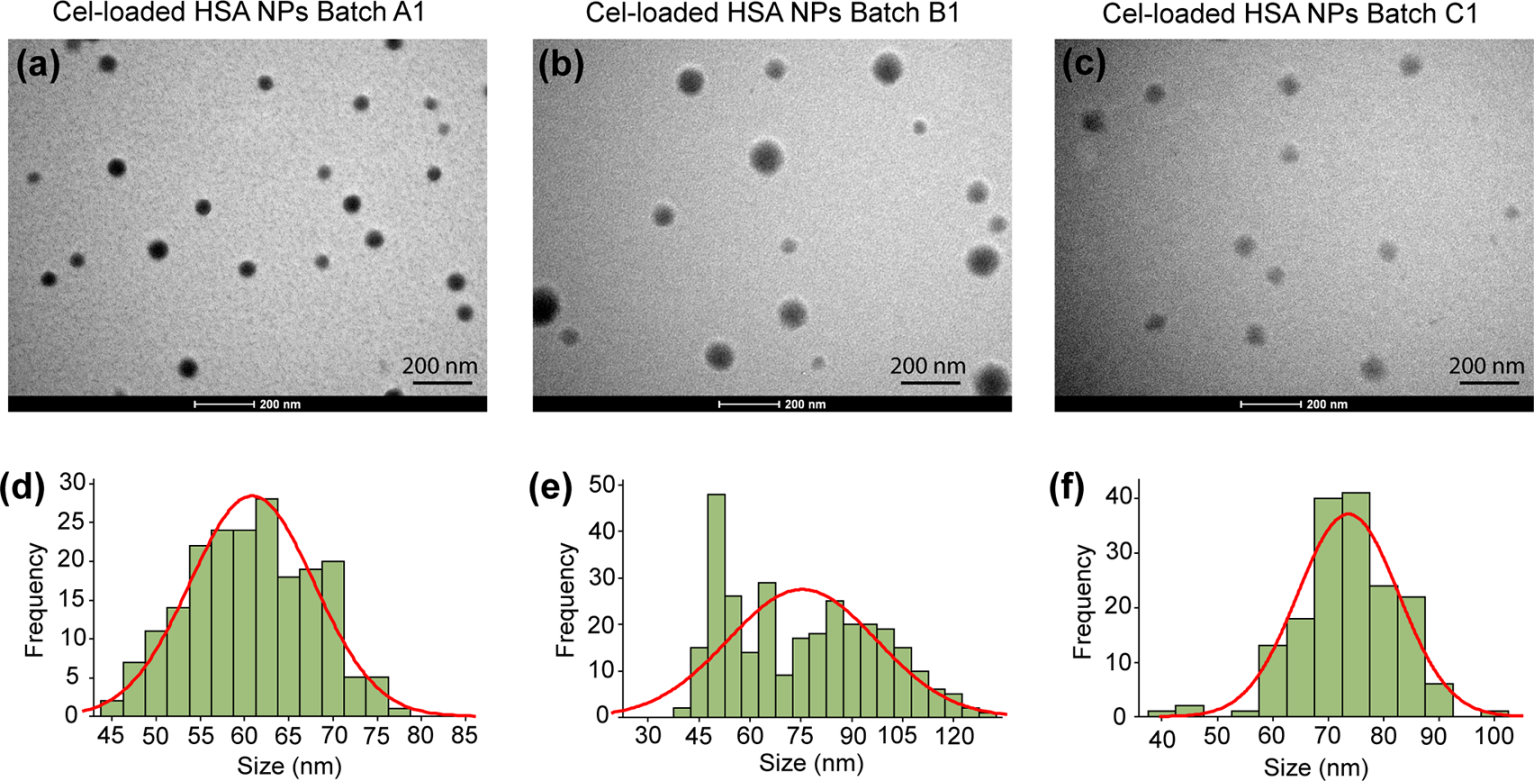
(a–c) TEM micrographs and (d–f) corresponding particle size distributions of three batches of Cel-loaded HSA NPs.

**Figure 2:**
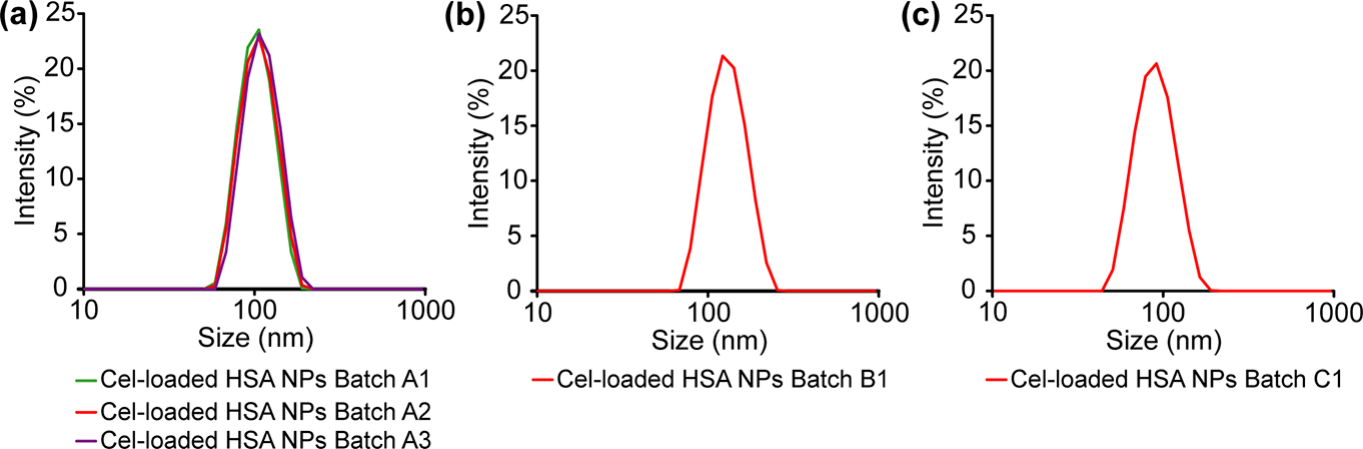
(a–c) Particle size distributions of five batches of Cel-loaded HSA NPs, as measured by DLS at 25 °C.

The size and morphology of the Cel-loaded HSA NPs were characterized using transmission electron microscopy (TEM), atomic force microscopy (AFM), and dynamic light scattering (DLS)-based particle size analysis. TEM micrographs (Figures 1a–c and S2a,b) of all the batches of Cel-loaded HSA NPs prepared using three different batches of starting HSA indicated that the NPs were spherical and non-agglomerated. The particle size distributions for all the batches of Cel-loaded HSA NPs are shown in Figures 1d–f and S2c,d. The average *d*_TEM_ of the Cel-loaded HSA NPs prepared using the same batch and using three different batches of starting HSA were 66 ± 5 nm (7%) and 72 ± 5 nm (7%), respectively (Table S2). Additionally, all the NPs in all batches have size <200 nm. AFM micrographs (Figure S3a,b) showed that the NPs were non-agglomerated. DLS-based particle size analysis of all the batches of Cel-loaded HSA NPs at pH ∼ 7.3 showed a single narrow particle size distribution curve (Figure 2a–c) with a relatively low PDI of <0.1 (Table S2), which indicated that the NPs were monodisperse. Such monodisperse particles can increase the efficacy of DDSs and reduce adverse reactions.^13^ The mean *Z*-average hydrodynamic diameters (*d*_hyd_) of the suspensions prepared using the same batch and using three different batches of starting HSA were 103 ± 3 nm (3%) and 105 ± 19 nm (18%), respectively (Table S2), which were larger than the size obtained by TEM as anticipated. The percentage relative standard deviation (%RSD) for both the above cases of *d*_hyd_ was <20%, which is within the acceptance criteria of US Pharmacopeia.^57^ The TEM and DLS data showed that our synthesis produced suitable average-sized Cel-loaded HSA NPs to penetrate the OA cartilage. The zeta-potential of all the five batches of Cel-loaded HSA NPs recorded at pH ∼ 7.3 was in the range −32 to −37 mV (Table S2), which indicated that the NPs were electrostatically stabilized against agglomeration.^58^ Overall, TEM, DLS-based particle size analysis, and zeta-potential results demonstrated that our synthesis method was highly reproducible.

### Encapsulation Efficiency and In Vitro Release of Cel from Cel-Loaded HSA NPs

The encapsulation efficiency (E.E.) of the HSA NPs for Cel was estimated using fluorescence spectroscopy, and it was 12%, i.e., 72 μg of Cel was loaded from a starting concentration of 600 μg. This corresponded to 72 μg/mL (188.79 μM) of Cel in the NP suspension (when Cel-loaded HSA NPs were redispersed in 1 mL of water after ultracentrifugation). This value is 91-fold higher than the concentrations of Cel (0.33–0.79 μg/mL, equivalent to 0.86–2.07 μM) that were detected in the synovial fluid of the arthritis patients after daily doses of 100 mg given twice orally for 5 days, which is a commonly used dose for arthritis patients.^59,60^ Thus, it is noteworthy that our formulation loaded Cel at a higher concentration than that required to induce a therapeutic response in the arthritic joint. E.E. was also estimated upon addition of a higher concentration of Cel (1200 μg) to the reaction medium, and it was 6.5%, corresponding to 78 μg/mL (204.52 μM). However, crystals were visible in the supernatant after the ultracentrifugation of the resultant NPs at this higher concentration. Thus, all of the studies were undertaken with NPs, where 600 μg of Cel was added to the reaction medium.

The in vitro cumulative release (C.R.) of Cel from NPs was studied in PBS in a volume of 60 mL at pH 7.4 by using fluorescence spectroscopy. For all three batches of Cel-loaded HSA NPs, there was a sustained release of Cel over 72 h, and the C.R. was 14–16% (Figure S4a–c), or 10.1–11.3 μg (Figure S4d–f), or 0.44–0.49 μM (Figure S4g–i). The surprisingly low C.R. could be because Cel has low solubility in aqueous medium and thus would be released in low concentrations. Moreover, the in vitro assay buffer was not analogous to synovial fluid, which is more complex than PBS.^27^ In vivo, NPs may be internalized by a range of cell types found in the joint, followed by gradual release of Cel, or the NPs may release Cel in the synovial fluid.

### Colloidal Stability of Cel-Loaded HSA NPs

**Figure 3:**
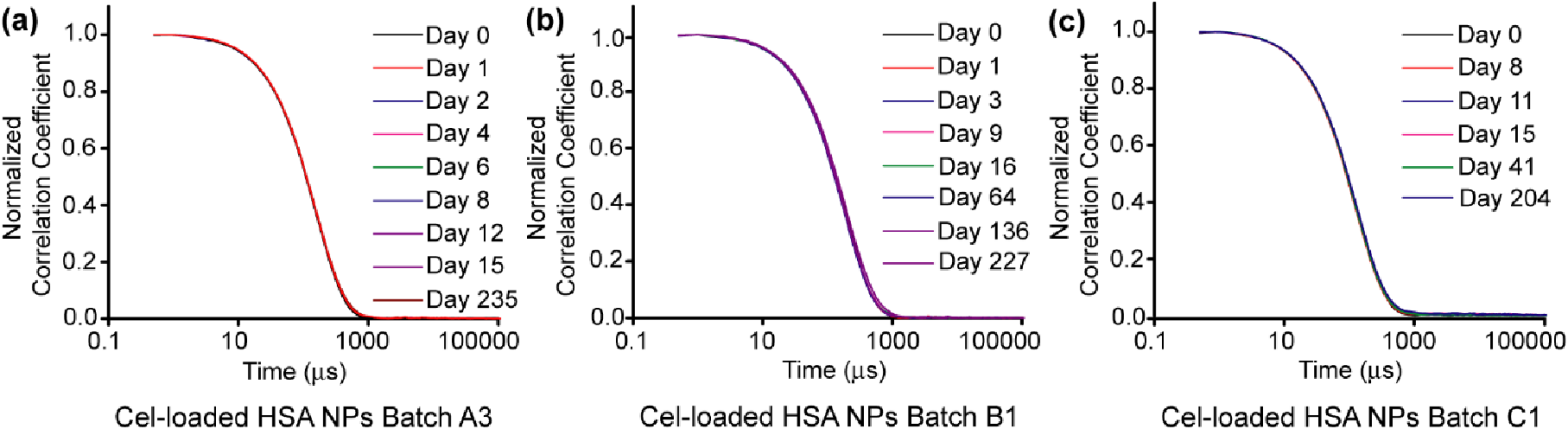
(a–c) DLS correlograms of three batches of Cel-loaded HSA NPs stored at 4 °C over time. The DLS measurements were carried out at 25 °C.

Colloidal stability of NP suspensions is an important parameter (a) to determine the optimum conditions for long-term storage and (b) to confirm stability for use for in vivo applications. For determining the storage conditions, Cel-loaded HSA NP suspensions were prepared using three different batches of starting HSA, and their colloidal stability was monitored at 4 °C (refrigeration condition) and 22 °C (controlled room temperature condition) over time by DLS. To confirm the absence of aggregates, DLS correlograms are presented. The correlograms for each batch of Cel-loaded HSA NP suspensions stored at 4 °C recorded on different days overlapped with no significant variations (Figure 3a–c), and the derived count rate was almost unchanged with a standard deviation ≤5% (Figure S5a–c) for at least 204 days, which demonstrates that the Cel-loaded HSA NP suspensions were stable (no significant change in particle size or number) for at least 204 days when stored at 4 °C. The absence of aggregation or loss of particles to sedimentation is critical for ensuring correct dosage in in vivo studies. For NP suspensions stored at 22 °C, correlograms for all the batches of Cel-loaded HSA NP suspensions recorded on different days overlapped with no significant variations for at least 15 days (Figure S6a–c), and the derived count rate was almost unchanged, with a standard deviation ≤5% (using the criterion established from storage at 4 °C), for at least 9 days (Figure S6d–f). After 15 days, correlograms for batch B1 recorded on different days overlapped with no significant variations (Figure S6b), but this was not the case for batch C1 (Figure S6c). However, in both the cases, the derived count rate decreased with the number of days, after 9 and 11 days for batches B1 and C1, respectively (Figure S6e,f), signifying loss of some NPs from the suspension. Thus, it can be concluded that NP suspensions when stored in refrigerator were stable for at least 6 months and when stored at controlled room temperature (RT) were stable for at least 9 days, demonstrating longer shelf life when stored in refrigerator.

For in vivo applications, the colloidal stability of Cel-loaded HSA NP suspensions prepared using two different batches of starting HSA was monitored at 37 °C over time by DLS. For both the batches, the correlograms recorded on different days until day 28 overlapped with no significant variations (Figure S7a,b), and the derived count rate was almost unchanged with a standard deviation ≤5% for 10 days (Figure S7c,d). After 10 days, the derived count rate started decreasing significantly (Figure S7c,d), indicating that the NP suspensions were stable for at least 10 days when incubated at 37 °C. This time period can be considered as sufficient time for the NPs to penetrate the extracellular matrix of OA cartilage and target chondrocytes after intra-articular injection at this temperature.^55^

### Internalization of Cel-Loaded HSA NPs by Primary Human Articular Chondrocytes from the Knee Joints of OA Patients

**Figure 4:**
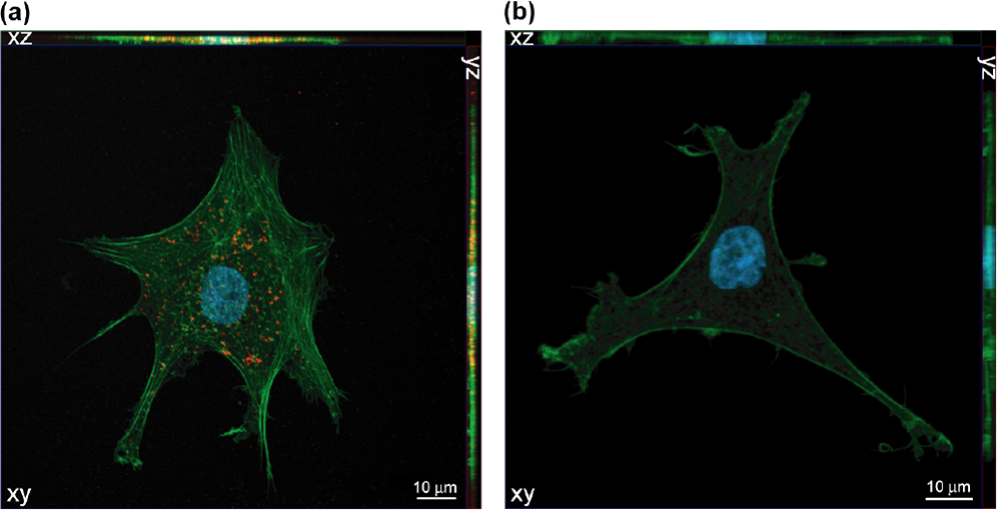
Orthogonal projections (*XY*, *XZ,* and *YZ*) of confocal images of hOAACs exposed for 4 h to (a) Alexa Fluor 568-labeled Cel-loaded HSA NPs (red) and (b) untreated control. Actin cytoskeleton was labeled with Alexa Fluor 488 phalloidin (green) and nuclei with DAPI (blue).

As the Cel-loaded HSA NPs were stable, monodisperse, and of a suitable size to penetrate OA cartilage, their capacity for internalization was studied in primary human articular chondrocytes from the knee joints of OA patients (hOAACs). For confirming internalization, the hOAACs (from one donor) were exposed to red Alexa Fluor 568-labeled Cel-loaded HSA NPs for 4 h and then observed by Airyscan microscopy with the highest possible resolution. Red fluorescence was visible inside the cells (Figure 4a and Video S1), which indicated that the labeled NPs were internalized and not attached on the surface of the hOAACs. As a control, untreated hOAACs were also observed, and no fluorescence was visible inside the cells (Figure 4b and Video S2).

### In Vitro Effects of Cel-Loaded HSA NPs on the Cellular Viability of hOAACs: MTS Assay

**Figure 5:**
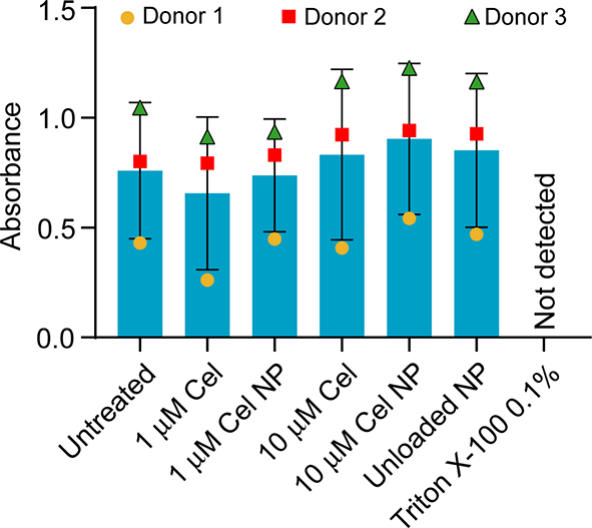
Effect of Cel-loaded HSA NPs exposure for 24 h on the cellular viability of hOAACs (*n* = 3 OA donors) as assessed by MTS assay. 1 and 10 μM Cel NP correspond to the concentrations of Cel-loaded HSA NPs calculated to contain these two concentrations of Cel. The concentration of unloaded control NP was equivalent to the concentration of NP in 10 μM Cel NP. Bars represent the mean ± SD of three independent biological experiments (one biological experiment for each donor), and the three data points per group represent the mean of each biological experiment. For each biological experiment, 3–4 technical replicates were obtained. For statistical analysis, a one-way ANOVA with Dunnett’s post-hoc test was used.

The metabolic status of hOAACs from three donors following exposure to Cel-loaded HSA NPs was assessed by the MTS assay, which measures the redox potential and is an indirect indicator of the cellular viability. Figure 5 shows that there was no significant difference in the redox potential of hOAACs treated with two different concentrations of Cel-loaded HSA NPs (containing 1 and 10 μM Cel) in comparison to the untreated control, indicating excellent biocompatibility of the Cel-loaded HSA NPs. Similar results were obtained for Cel and unloaded NPs (Figure 5). The redox potential of hOAACs was minimal in the Triton X-100 positive control group, indicating the validity of the assay.

### In Vitro Effects of Cel-Loaded HSA NPs on Prostaglandin E_2_ Levels of hOAACs

**Figure 6:**
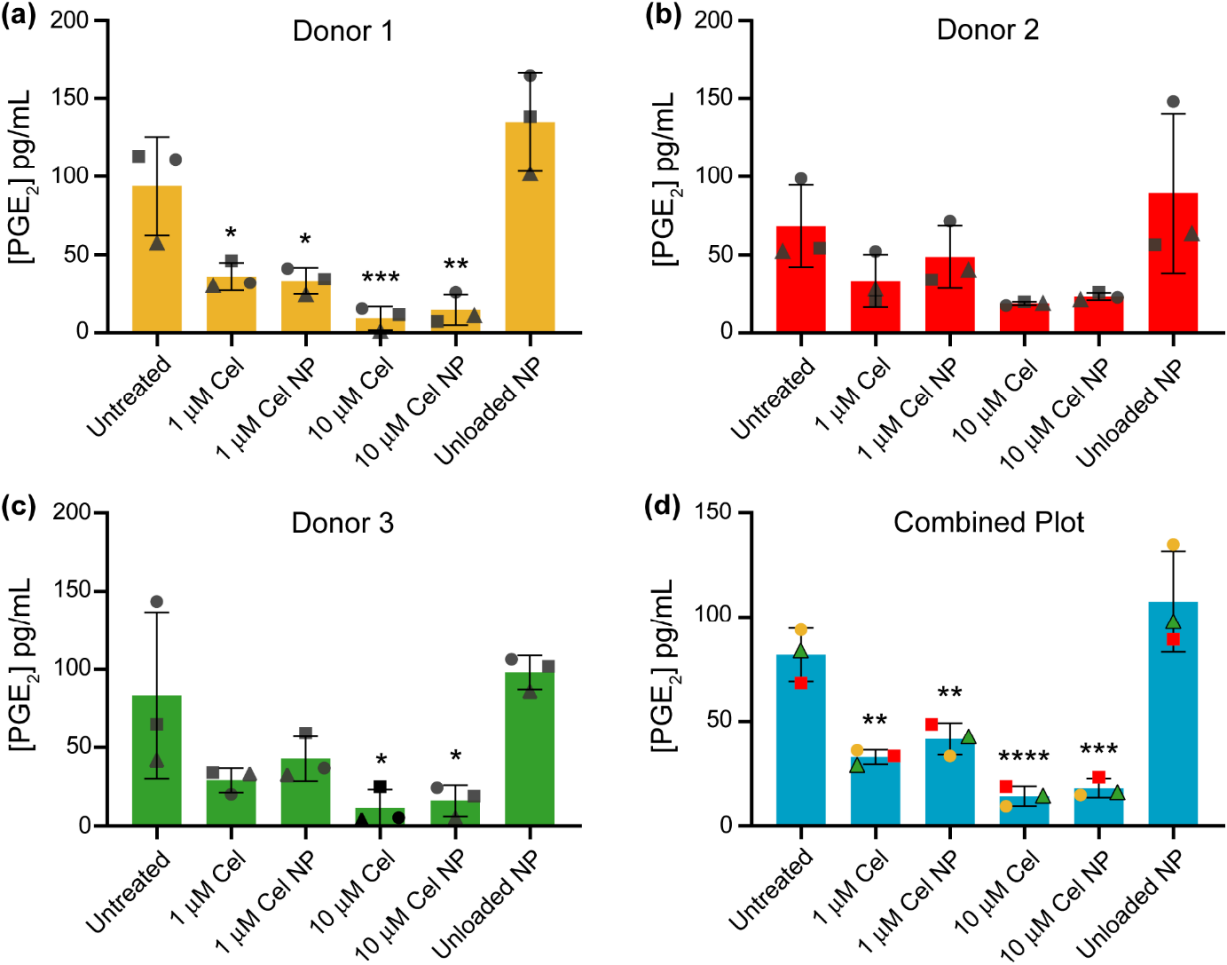
(a–d) Reduction in PGE_2_ concentrations released by hOAACs by both Cel and Cel-loaded HSA NPs. 1 and 10 μM Cel NP correspond to the concentrations of Cel-loaded HSA NPs calculated to contain these two concentrations of Cel. The concentration of unloaded control NP used was equivalent to the concentration of NP in 10 μM Cel NP. For parts a–c, bars represent the mean ± SD of three biological experiments for each donor, and the three data points per group represent the mean of each biological experiment. For part d, bars represent the mean ± SD of the three donors, and the three data points per group represent the mean of three biological experiments for each donor. For each biological experiment, 2 or 3 technical replicates were done. For statistical analysis, a one-way ANOVA with Dunnett’s post-hoc test was used. *p < 0.05; **p < 0.01; ***p < 0.001; ****p < 0.0001 compared to untreated control.

The potential anti-inflammatory activity of Cel-loaded HSA NPs was evaluated in hOAACs from the knees of three OA donors. Cel inhibits the activity of COX-2 enzymes, which are primarily responsible for the formation of prostaglandin E_2_ (PGE_2_), a key mediator of inflammation. The concentrations of PGE_2_ were measured in hOAACs treated with and without Cel-loaded HSA NPs. Basal levels of PGE_2_ were sufficiently high in the hOAACs cells, and as such, no additional inflammatory stimulators were required. Figure 6a shows that Cel and Cel-loaded HSA NPs at two drug concentrations significantly reduced the concentrations of PGE_2_ released by hOAACs from donor 1, whereas unloaded NPs had no effect. A similar trend was observed in hOAACs from donor 2 (Figure 6b) and donor 3 (Figure 6c). The combined plot of the three donors (Figure 6d) showed a significant lowering of PGE_2_ concentrations in the hOAACs exposed to Cel and Cel-loaded HSA NPs compared to that in untreated control NPs. Thus, it can be concluded that the Cel-loaded HSA NPs have anti-inflammatory property, and this was due to the Cel loaded in the NPs. Moreover, these results indicated that the method of synthesizing the NPs did not alter the activity of Cel, and that the Cel must have been released from NPs inside the hOAACs where it then inhibited the COX-2 activity.

Note that we used 1 μM and 10 μM Cel for in vitro studies, covering both a concentration within the therapeutic range (0.86–2.07 μM) for arthritis patients^59,60^ and a concentration approximately five times higher to check the biocompatibility and efficacy of a high concentration in cell bioassays. Both concentrations displayed excellent biocompatibility and reduced the PGE_2_ concentrations secreted from hOAACs.

### In Vitro Effects of Cel-Loaded HSA NPs on Cellular Viability and PGE_2_ Levels of Lipopolysaccharide (LPS)-Stimulated Human Leukemia Monocytic THP-1 Cells

As monocytes play an important role in the pathogenesis of OA,^61^ we also studied the effect of Cel-loaded HSA NPs on the cellular viability and release of PGE_2_ from LPS-stimulated THP-1 cells. THP-1 cells were stimulated with LPS in order to mimic the inflammatory state such as that found in hOAACs. Figure S8 indicates that Cel-loaded HSA NPs, Cel, and unloaded HSA NPs did not reduce the redox potential of LPS-stimulated THP-1 cells according to the MTS assay (data shown only for the highest concentrations used). These data validate the responses seen in the hOAACs.

Finally, the effect of Cel-loaded HSA NPs on the PGE_2_ levels released from LPS-stimulated THP-1 cells was assessed. The data in Figure S9 indicate that stimulating the THP-1 cells with 1 μg/mL LPS increased the PGE_2_ concentration. When THP-1 cells were pretreated with four different concentrations of Cel for 120 min and then stimulated with 1 μg/mL LPS, the released PGE_2_ concentrations were significantly decreased compared with cells stimulated with LPS only. Although Cel-loaded HSA NPs having low concentrations of 0.01 and 0.1 μM Cel did not reduce the PGE_2_ concentrations released from LPS-stimulated THP-1 cells, NPs loaded with 1 and 10 μM of Cel significantly did so. This difference in sensitivity to Cel released from the NPs could be due to a suboptimal release profile for Cel, where the kinetics from the two lower concentrations may not have reached the threshold for enzyme inhibition. Overall, the experiment in THP-1 cells demonstrated that the NPs could release Cel in concentrations required to reduce the PGE_2_ levels, and the data were consistent with the bioactivity data obtained in hOAAC cells.

## Conclusion

In conclusion, we have developed a method for synthesizing Cel-loaded HSA NPs, which is reproducible, economical, requires less stringent process controls, and uses materials approved by the regulatory agencies. The method developed the colloidally stable Cel-loaded albumin NPs, without using chemical protein cross-linkers, polymer coatings, oils, Class 1/2 solvents, or temperatures at which denaturation of albumin becomes a factor. The NPs were spherical, nonagglomerated, monodisperse, biocompatible, and bioactive in vitro. The NPs could load Cel at concentrations well in excess of that required to elicit therapeutic responses in the arthritic joint. The NPs were internalized by hOAACs, and the released Cel reduced the concentration of cell released PGE_2_. The small particle size renders them suitable for penetrating the OA cartilage. They are therefore the first albumin-based NP agent reported that addresses all known criteria for successful IA delivery to treat OA. The approach may reduce or avoid systemic side-effects associated with orally administered Cel by localizing it to the joint. Our method can potentially be used to load other drugs in albumin NPs to treat other diseases by a range of routes of administration.

## Experimental Section

### Materials

Albumin from human serum (lyophilized powder, essentially fatty acid free), albumin from human serum (lyophilized powder, ≥96%, agarose gel electrophoresis), sodium hydroxide, celecoxib ≥98% (HPLC), acetone, pepsin from porcine gastric mucosa (lyophilized powder, ≥3,200 units/mg protein), hydrochloric acid, dichloromethane, acetic acid, lipopolysaccharides from *Escherichia coli* O55:B5 (γ-irradiated, BioXtra, suitable for cell culture), dexamethasone powder, Dulbecco′s Modified Eagle′s Medium – low glucose, Pronase from *Streptomyces griseus*, fibroblast growth factor 2, Dulbecco′s Modified Eagle′s Medium/Nutrient Mixture F-12 Ham, trypsin-EDTA solution, sodium pyruvate solution 100 mM, l-ascorbic acid 2-phosphate sesquimagnesium salt hydrate, PBS tablets, sodium bicarbonate powder, Mowiol 4–88, Triton X-100 (Sigma-Aldrich), RPMI medium 1640 (1×) + GlutaMAX-1, fetal bovine serum, penicillin-streptomycin 10,000 U/mL, collagenase type II powder, MEM non-essential amino acids solution 100×, insulin-transferrin-selenium-ethanolamine 100×, Opti-MEM I reduced serum medium (Gibco), PGE_2_ ELISA kit monoclonal, ultrapure water (Cayman Chemical Company), CellTiter 96 aqueous one solution cell proliferation assay (Promega), Water – CHROMASOLV Plus for HPLC (Honeywell), Alexa Fluor 568 NHS ester, DMSO anhydrous, Hoechst 33342 (trihydrochloride, trihydrate, 10 mg/mL solution in water), Alexa Fluor 488 phalloidin (Invitrogen), formaldehyde fixative 16% (w/v) ultrapure EM grade MaxTag Histo for IHC (Rockland Immunochemicals), Sil 180 silicone oil bath liquid (Thermo Scientific), and ethanol absolute (Lennox) were purchased and used without any purification.

Spectra-Por Float-A-Lyzer G2, black, 1 mL, MWCO 3.5–5 kDa (Spectrum Laboratories, Inc.), 50 mL Gastight Syringe Model 1050 TLL, PTFE Luer Lock (Hamilton Company), tube Quick-Seal, polypropylene, 2.0 mL, 11 × 32 mm (Labplan), tube Quick-Seal, polypropylene, 8 mL, 16 × 57 mm (Labplan), PTFE syringe tubing, gauge 16, L 24 in. (Sigma-Aldrich), and syringe infusion pump (KDS 200, KD Scientific) were also purchased.

### Optimized Synthesis of Cel-Loaded HSA NPs

In a 100 mL round-bottom (RB) flask, 100 mg of HSA (essentially fatty acid free) was weighed, and 10 mL of water was added. Then the flask was kept in an oil bath maintained at 30 °C by using a heating magnetic stirrer. The solution was stirred at ∼450 rpm. After albumin was completely dissolved, freshly prepared NaOH solution was added to maintain the pH at ∼8.1, and the solution was stirred for 1 h. Then, 60 μL of a 10 mg/mL Cel solution in ethanol was added to it. Following this, the solution was stirred for another 2 h. Then, 50 mL of acetone was added (while the solution was stirred at ∼450 rpm) using a syringe infusion pump, a Hamilton syringe, and PTFE syringe tubing at a rate of 1 mL/min. After the addition of acetone, the RB was stoppered; the temperature of the oil bath was raised to 50 °C, and the NP suspension was stirred for another 16 h. After 16 h, the acetone was evaporated by using the rotary evaporator at 50 °C and 100 mb. Then, the suspension was ultracentrifuged in polypropylene tubes at 35,000 rpm and 4 °C for 30 min. The obtained pellets were resuspended in water and again ultracentrifuged under the same conditions. Finally, the pellets obtained (from one reaction) were resuspended in 8 mL of water, and the NP suspension was stored in the refrigerator. The pH of the Cel-loaded NP suspension was ∼7.3. The unloaded HSA NPs were also prepared using the same method but with ethanol instead of Cel in ethanol.

To check the reproducibility of the synthesis method, different batches of Cel-loaded HSA NPs were prepared using three different batches of starting HSA. The HSA batches used were SLBM7779V, SLCB2530, and SLCF6784. Cel-loaded HSA NPs prepared from SLBM7779V batch of HSA are referred to as batches A1, A2, A3, etc., SLCB2530 batch of HSA are referred to as batches B1, B2, and SLCF6784 batch of HSA are referred to as batches C1, C2. All characterizations were carried out using NPs prepared using the SLBM7779V batch of HSA. However, to check the reproducibility of the synthesis method, TEM and DLS were also carried out for the Cel-loaded NPs prepared using SLCB2530 and SLCF6784 batches of HSA.

To obtain the optimized conditions mentioned above, Cel-loaded HSA NPs were synthesized using different experimental conditions, as mentioned in Table S1.

### Characterization of Cel-Loaded HSA NPs

NPs were characterized using fluorescence spectroscopy, TEM, AFM, and DLS-based particle size analysis. For fluorescence spectroscopy, the suspensions of Cel-loaded HSA NPs and unloaded HSA NPs (both prepared using SLBM7779V batch of HSA) were diluted with water, and their emission spectra were recorded using Varian Cary Eclipse fluorescence spectrophotometer. The excitation wavelength was set at 242 nm, and excitation/emission slit widths were fixed at 10 nm. For TEM, a Cel-loaded HSA NP suspension was diluted with water, and a drop was placed onto carbon-coated copper TEM grids (made hydrophilic using PELCO easiGlow Glow Discharge Cleaning System before sample preparation). After 3 min, the excess sample was blotted using filter paper. The grids were dried overnight at RT and then imaged using FEI Tecnai G2 12 BioTWIN transmission electron microscope at an accelerating voltage of 80 kV. For each sample, at least 100 NPs were imaged and analyzed using ImageJ and Minitab software. TEM was carried out for different batches of Cel-loaded HSA NPs prepared using SSLBM7779V, SLCB2530, and SLCF6784 batches of starting HSA.

For AFM, prior to sample preparation, the mica disc was cleaved and ozone-UV treated for 2 min. Then, 5 μL of diluted Cel-loaded HSA NP suspension (prepared using SLBM7779V batch of HSA) was placed on the mica disc and allowed to dry at RT. After drying, the sample was imaged using an MFP-3D atomic force microscope from Asylum Research, Oxford Instruments in amplitude modulation mode in air at RT with an NCH probe (Nanosensors) with a nominal spring constant of 42 N/m and resonant frequency of 320 kHz.

For DLS, Cel-loaded HSA NP suspensions (prepared using 3 batches of starting HSA) were diluted with water, and their particle size and zeta-potential were recorded using Zetasizer Nano ZS (Malvern Instruments) at 25 °C. For each sample, 3 measurements were carried out.

### E.E. and In Vitro Release of Cel from Cel-Loaded HSA NPs

For calculating E.E., pellets of Cel-loaded HSA NPs (from one reaction) after ultracentrifugation were resuspended in 2 mL of water. From this, 50 μL of NP suspension was mixed with 1500 μL of 3 mg/mL pepsin solution in 0.015 N HCl. The pH of the resulting solution was ∼2.0. Then, the solution was incubated at 37 °C overnight. After incubation, Cel was extracted in 2.5 mL of dichloromethane (DCM), and its emission spectrum was recorded using the excitation wavelength of 242 nm, excitation slit width of 10 nm, and emission slit width of 10 nm. The emission intensity at 364 nm was noted and using the calibration curve of different concentrations of Cel in DCM (Figure S10), the concentration of extracted Cel was calculated. This corresponds to the weight of extracted Cel in 1 mL of DCM. The weight of Cel extracted in 2.5 mL of DCM was calculated, which is equivalent to the weight of Cel encapsulated in 50 μL of the NP suspension. From this, the weight of Cel encapsulated in 2 mL of the NP suspension was calculated. Finally, the E.E. was calculated using the following formula:
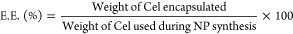
1

Two batches of Cel-loaded HSA NPs (prepared from SLBM7779V) were analyzed, and for each batch, four repeats were carried out. For both batches, separate calibration curves were made. E.E. was also calculated for a batch of Cel-loaded HSA NPs (prepared from SLBM7779V), where 60 μL of 20 mg/mL Cel was added to the reaction medium instead of 60 μL of 10 mg/mL Cel.

For in vitro release, first the dialysis device was washed with 10% ethanol, followed by water based on the manufacture’s protocol. Then, pellets of Cel-loaded HSA NPs (from one reaction) after ultracentrifugation were resuspended in 1 mL of PBS of pH 7.4 and taken in Float-A-Lyzer G2 Dialysis Device, MWCO 3.5–5 kDa. Following this, the dialysis device was inserted in a glass bottle containing 60 mL of PBS. The glass bottle was kept in a shaking incubator maintained at 1050 rpm and 37 °C. At time point 0 h, 1 mL of the release medium was taken from the glass bottle, and 1 mL of fresh PBS was added back to the glass bottle. Similarly, 1 mL of the release media was taken at different time points up to 72 h, with fresh PBS added at each time point. The emission spectra of all the release media taken out were recorded using an excitation wavelength of 242 nm, excitation slit width of 10 nm, and emission slit width of 10 nm. The emission intensities at 405 nm were noted. Using the calibration curve of Cel in PBS, the concentrations of Cel released at different time points were calculated. From this, the C.R. (in μg and μM) was calculated, and using the formula below, the C.R. (%) was calculated.
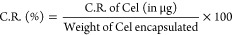
2

Finally, C.R. in – %, μg, and μM was plotted against time. Three batches of Cel-loaded HSA NPs (prepared from SLBM7779V) were analyzed, and for each batch, separate calibration curves were made.

### Colloidal Stability of Cel-Loaded HSA NPs

For storage, the colloidal stability of Cel-loaded HSA NPs in water at pH 7.3 was evaluated at 4 and 22 °C. For this, the suspension of Cel-loaded HSA NPs was diluted with water, and its particle size was recorded at 25 °C using the DLS on day 0. Then, the suspension was divided into two parts, one part was stored at 4 °C (refrigerator), and the other part at 22 °C (controlled RT). Next day (i.e., day 1), both the NP suspensions were taken out from 4 and 22 °C, allowed to equilibrate to RT, and then the particle size of the NP suspensions was recorded using DLS at 25 °C. Similarly, NP suspensions were taken out from 4 and 22 °C on different days (for at least 204 days), and their particle sizes were recorded using DLS at 25 °C. Finally, the correlograms and derived count rates were compared and analyzed. Cel-loaded HSA NPs prepared using three batches of starting HSA were analyzed.

For in vivo applications, the colloidal stability of Cel-loaded HSA NPs in water at pH 7.3 was evaluated at 37 °C. For this, the suspension of Cel-loaded HSA NPs was diluted with water, and its particle size was recorded at 25 °C using DLS on day 0. Then, the suspension was kept in an incubator maintained at 37 °C. Next day (i.e., day 1), the NP suspension was taken out from the incubator and allowed to equilibrate to RT for around 15 min, and then the particle size of the NP suspension was recorded using DLS at 25 °C. Similarly, the NP suspension was removed from 37 °C incubator on different days (up to 28 days), and the particle size was recorded using DLS at 25 °C. Finally, the correlograms and derived count rates were compared and analyzed. Cel-loaded HSA NPs prepared using two batches of starting HSA (SLCB2530 and SLCF6784) were analyzed.

### Primary Cell Culture of hOAACs

Primary human articular chondrocytes were obtained from surgical off-cuts of three OA patients (hOAACs) undergoing total knee arthroplasty (TKA) at the National Orthopaedic Hospital, Cappagh (NOHC), Dublin, Ireland. Each patient provided fully informed consent under Ethics License number: Capp/2019/ETH/SH-CEO-234. Once tissues had been isolated, cartilage was dissected and digested in a 0.2% (w/v) Pronase solution in Dulbecco’s Modified Eagle’s Medium (DMEM) supplemented with 1% (v/v) sodium pyruvate and 1% (v/v) Pen/Strep for 2 h at 37 °C with agitation. Following this, Pronase solution was replaced by a 0.075% (w/v) collagenase II-DMEM solution supplemented with 1% (v/v) Pen/Strep, 10% (v/v) FBS, 10 μM l-ascorbic acid 2-phosphate, and 5 ng/mL fibroblast growth factor 2 (FGF-2) and incubated for 14–16 h at 37 °C. Digested cartilage solutions were passed through a 70 μm cell strainer, and hOAACs pelleted through centrifugation at 350 rcf for 5 min. The supernatant was then removed, hOAACs resuspended in DMEM/F-12 Ham 1:1 mixture containing l-glutamine supplemented with 10% (v/v) FBS, 1% (v/v) insulin-transferrin-selenium-ethanolamine (ITS-X), 10 μM l-ascorbic acid 2-phosphate, 1% (v/v) non-essential amino acids, 0.11 mg/mL sodium pyruvate, and 1% (v/v) Pen/Strep (referred to as expansion media), counted, and seeded into cell culture flasks. hOAACs were expanded in culture in expansion media and maintained in a humidified tissue culture incubator containing 5% CO_2_ at 37 °C. Passages 2–6 of the hOAACs were used in subsequent experiments.

### Culture of THP-1 Monocytes

THP-1 cell line (ATCC TIB-202) was obtained from the Conway Institute of Biomolecular and Biomedical Research, University College Dublin as a gift and cultured in RPMI-1640-GlutaMAX media supplemented with 10% (v/v) fetal bovine serum (FBS) and 1% (v/v) penicillin-streptomycin (Pen/Strep) (referred as full media). The cell line was maintained in a humidified tissue culture incubator containing 5% CO_2_ at 37 °C. For studying the effect of Cel-loaded HSA NPs on the cellular viability and PGE_2_ levels of LPS-stimulated THP-1 cells, RPMI-1640-GlutaMAX media supplemented with 0.1% (v/v) FBS and 1% (v/v) Pen/Strep (referred to as reduced media) were used.

### Confocal Imaging of hOAACs Treated with Labeled Cel-Loaded HSA NPs

Cel-loaded HSA NPs were labeled with Alexa Fluor 568 dye. 100 μL of NP suspension (pellet redispersed in 1.2 mL of water after ultracentrifugation) was mixed with 900 μL of 0.1 M sodium bicarbonate buffer (pH 8.2). Then, 25 μL of the fresh Alexa Fluor 568 NHS ester solution (10 mg/mL in anhydrous dimethyl sulfoxide) was slowly added to the NP suspension, and the mixture was stirred at ∼450 rpm for 1 h. After this, the resulting suspension was ultracentrifuged in polypropylene tubes at 35,000 rpm and 4 °C for 30 min, and the resulting pellet was resuspended in water and again ultracentrifuged. Finally, this pellet was resuspended in 1 mL of water and used for treating the cells.

For confocal/Airy imaging, 45,000 hOAACs/well were seeded onto individual coverslips placed inside a 12 well plate and grown overnight. Then, the expansion media were removed, and the hOAACs were washed with PBS. Following this, Opti-MEM media (with 0.1% FBS) containing Alexa Fluor 568-labeled Cel-loaded HSA NPs (having ∼10 μM of Cel) were added to the hOAACs. Untreated hOAACs in three wells were used as the controls. After 4 h, the conditioned medium was aspirated, and the hOAACs were washed with PBS twice. Then, the hOAACs were fixed with 4% formaldehyde solution in PBS for 15 min at RT. After this, the hOAACs were washed with PBS twice. Then, the hOAACs were permeabilized with 0.1% (v/v) Triton X-100 for 5 min at RT. Again, the hOAACs were washed twice with PBS. Finally, the actin cytoskeleton and nucleus of the hOAACs were stained. The actin cytoskeleton was stained with Alexa Fluor 488 phalloidin (1/400 in PBS) for 30 min at RT, and the nucleus was stained with DAPI (1/2000 in PBS) for 5 min at RT. Following each staining, the hOAACs were washed with PBS twice, and finally the coverslips with hOAACs were mounted on glass microscopic slides with a drop of the mounting agent, Mowiol. The slides were allowed to cure for 1 h at RT and then stored in a refrigerator. The samples were imaged using a Zeiss LSM800 Airyscan microscope, with the 63×/NA 1.4 PlanApo oil lens. For excitation, diode lasers with wavelengths 405, 488, and 561 nm were used. Airy processing, maximal intensity ortho *XYZ* projections, and export in TIFF format were performed with original Zeiss ZEN software under careful adjustment of all parameters. All data were acquired with the same lasers, detectors, pixel size, and pixel dwell time parameters and normalized before exporting images and videos. No additional image processing was applied. Videos were made with Imaris 9.8 software.

### In Vitro Effects of Cel-Loaded HSA NPs on the Cellular Viability of hOAACs: MTS Assay

To determine the effect of Cel-loaded HSA NPs on the cellular viability of hOAACs, MTS assays were carried out. Cells were seeded at a density of 45,000 cells/well in 12 well plates and grown overnight in expansion media. Then, the media were removed, and the hOAACs were washed with PBS. Following this, Opti-MEM media (supplemented with 0.1% FBS) containing two concentrations of Cel (1 and 10 μM), two concentrations of Cel-loaded HSA NPs (containing 1 and 10 μM of Cel as calculated by E.E.), unloaded HSA NPs (equivalent to the concentration of NP in 10 μM Cel NP), and Triton X-100 (0.1%, v/v) were added. After 24 h incubation, treatment media were aspirated, and the hOAACs were washed with PBS. Finally, the MTS assay was carried out according to the manufacturer’s protocol. The MTS assays were carried out in hOAACs from three donors. For each donor, one biological experiment was carried out.

### In Vitro Effects of Cel-Loaded HSA NPs on the Cellular Viability of LPS-Stimulated THP-1 Monocytes: MTS Assay

1 ×10^5^ THP-1 cells were seeded per well in a 96 well plate. The cells were pretreated with four concentrations of free Cel (0.01 μM, 0.1 μM, 1 μM, and 10 μM), four concentrations of Cel-loaded HSA NPs (containing 0.01 μM, 0.1 μM, 1 μM, and 10 μM of Cel), unloaded HSA NPs (equivalent to the concentration of NP in 10 μM Cel NP), and Triton X-100 (0.05% v/v). After 2 h, the cells were stimulated with 1 μg/mL of LPS and incubated for 24 h. As a control, THP-1 cells were incubated with LPS alone. Following this, the cells were centrifuged at 450 rcf at RT for 5 min. Then, the supernatant media were aspirated, and the MTS assay was carried out according to the manufacturer’s protocol. For each sample, two separate biological experiments were carried out.

### In Vitro Effects of Cel-Loaded HSA NPs on PGE_2_ Levels of hOAACs and LPS-Stimulated THP-1 Cells

Anti-inflammatory marker activity of Cel-loaded HSA NPs was assessed in hOAACs and THP-1 cells. For hOAACs, 45,000 cells/well were seeded in a 12 well plate and grown overnight. Then, the expansion media were removed, and the hOAACs were washed with PBS. Following this, Opti-MEM media (with 0.1% FBS) containing two concentrations of Cel (1 and 10 μM), two concentrations of Cel-loaded HSA NPs (containing 1 and 10 μM of Cel as calculated by E.E.), and unloaded HSA NPs (equivalent to the concentration of NP in 10 μM Cel NP) were added to the hOAACs. After 24 h incubation, conditioned media were collected and stored at −80 °C. Later, a PGE_2_ ELISA was carried out in conditioned media according to the manufacture’s protocol. The undiluted samples were used for ELISA, and the standard curve was made in Opti-MEM media supplemented with 0.1% FBS. The data obtained were analyzed using the four parameter logistic (4PL) curve fit tool from the “MyAssays” website, and then the results were plotted in GraphPad Prism. The experiment was carried out in hOAACs from three donors, and in each case, three separate biological experiments were carried out.

For THP-1 cells, 1 × 10^5^ cells/well were seeded in a 96 well plate. Cells were pretreated with the same groups as for the MTS assay in these cells (except Triton X-100) and incubated for 2 h. Following this, the cells were stimulated with 1 μg/mL of LPS and incubated for another 24 h. As a control, THP-1 cells without any treatment were stimulated with LPS. After 24 h, the cells were centrifuged at 4000 rcf at 4 °C for 10 min, and the conditioned media were collected and stored at −80 °C. PGE_2_ ELISA was carried out in conditioned media following the manufacturer’s protocol. The undiluted samples were used for ELISA, and the standard curve was made in reduced media. Data obtained were analyzed by using the 4PL curve fit tool and plotted in GraphPad Prism. For each sample, three separate biological experiments were carried out.

### Statistics

A one-way ANOVA with Dunnett’s post-hoc test was used to compare groups with untreated controls for hOAACs and with “LPS-stimulated only” for the THP-1 cells.

## Supporting Information Available

Multiple conditions used to prepare the Cel-loaded HSA NPs, along with TEM sizes tabulated; fluorescence spectra of unloaded and Cel-loaded HSA NPs; TEM micrographs and corresponding particle size distributions of two additional batches of Cel-loaded HSA NPs synthesized using the starting HSA batch SLBM7779V; AFM micrographs of Cel-loaded HSA NPs; TEM sizes, DLS-based hydrodynamic diameters, PDI, and zeta potential of different batches of Cel-loaded HSA NPs synthesized using the three different batches of starting HSA (SLBM7779V, SLCB2530, and SLCF6784) tabulated; cumulative release of Cel from three batches of Cel-loaded HSA NPs synthesized using the starting HSA batch SLBM7779V in PBS at pH 7.4 and 37 °C over 74 h; derived count rates (recorded on different days) of three batches of Cel-loaded HSA NPs (maintained at 4 °C) synthesized using three different batches of starting HSA (SLBM7779V, SLCB2530, and SLCF6784); correlograms and derived count rates (recorded on different days) of three batches of Cel-loaded HSA NPs (maintained at 22 °C) synthesized using the three different batches of starting HSA (SLBM7779V, SLCB2530, and SLCF6784); correlograms and derived count rates (recorded on different days) of two batches of Cel-loaded HSA NPs (maintained at 37 °C) synthesized using the two different batches of starting HSA (SLCB2530 and SLCF6784); effect of Cel-loaded HSA NPs on the cellular viability of LPS-stimulated THP-1 cells; ELISA results showing the reduction in PGE_2_ concentrations released by LPS-stimulated THP-1 cells by both Cel- and Cel-loaded HSA NPs; and fluorescence calibration curves used for calculating the E.E. (PDF)
Volume reconstruction from z-stack confocal images of hOAACs exposed for 4 h to Alexa Fluor 568-labeled Cel-loaded HSA NPs (MP4)
Volume reconstruction from z-stack confocal images of untreated hOAACs (MP4)
