# Supplementary material for: Reproducible Synthesis of Biocompatible Albumin Nanoparticles Designed for Intra-articular Administration of Celecoxib to Treat Osteoarthritis
Source: ACS Appl Mater Interfaces. 2024 Mar 14;16(12):14633–44. doi: 10.1021/acsami.4c02243 (PMC10982941; doi:10.1021/acsami.4c02243)
Supplement: Supplementary file 1 — am4c02243_si_001.pdf [file am4c02243_si_001.pdf]

Supporting Information

Reproducible Synthesis of Biocompatible Albumin Nanoparticles Designed for Intra-articular Administration of Celecoxib to treat Osteoarthritis

Rumi Khandelia,[†,⊥] Tom Hodgkinson,[‡] Daniel Crean,[†⊥] Dermot F. Brougham,[§] Dimitri Scholz,[⊥] Hossam Ibrahim,[δ,⊥] Susan J. Quinn[§], Brian J. Rodriguez,[δ,⊥] Oran D. Kennedy,[‡] John M. O'Byrne[#], and David J. Brayden [†,⊥,*]

[†]UCD School of Veterinary Medicine, University College Dublin, Belfield, Dublin D04 V1W8, Ireland.

[§]UCD School of Chemistry, University College Dublin, Belfield, Dublin D04 V1W8, Ireland.

[δ]UCD School of Physics, University College Dublin, Belfield, Dublin D04 V1W8, Ireland.

[⊥]UCD Conway Institute, University College Dublin, Belfield, Dublin D04 V1W8, Ireland.

[‡]Department of Anatomy and Regenerative Medicine, Royal College of Surgeons in Ireland, 123 St. Stephen's Green, Dublin D02 YN77, Ireland.

[#]National Orthopaedics Hospital, Cappagh, Dublin D11 EV29, Ireland.

Correspondence Email: david.brayden@ucd.ie

Table S1. Different conditions used to prepare the Cel-loaded HSA NPs along with d_{TEM}.

	**Condition used (d_{TEM} in nm)**			
**Different concentrations of HSA used**	1% (75.4)	5% (106.7)[1]		
**Different purity levels of HSA used**	Normal HSA (85.7)	Essentially fatty acid free HSA (75.4)		
**Different stabilization temperatures used**	30 °C (108.2)<br>The NPs were not stable	50 °C (75.4)		
**Different incubation time used at 50 °C**	3 h (107.1)	6 h (91.1)	12 h (96.4)	16 h (75.4)
**Different pH used**	~6.0 (363.8)[1]<br>Many NPs were larger than 200 nm	~7.0 (76.1)<br>Some NPs were larger than 200 nm	~8.1 (75.4)	

[1]Acetone used for 5% HSA and at 6.0 pH was 15 mL.

Note - For all the individual data point, rest of the conditions used were same as optimum condition and all the batches of Cel-loaded HSA NPs were synthesized using the starting HSA batch SLCB2530.

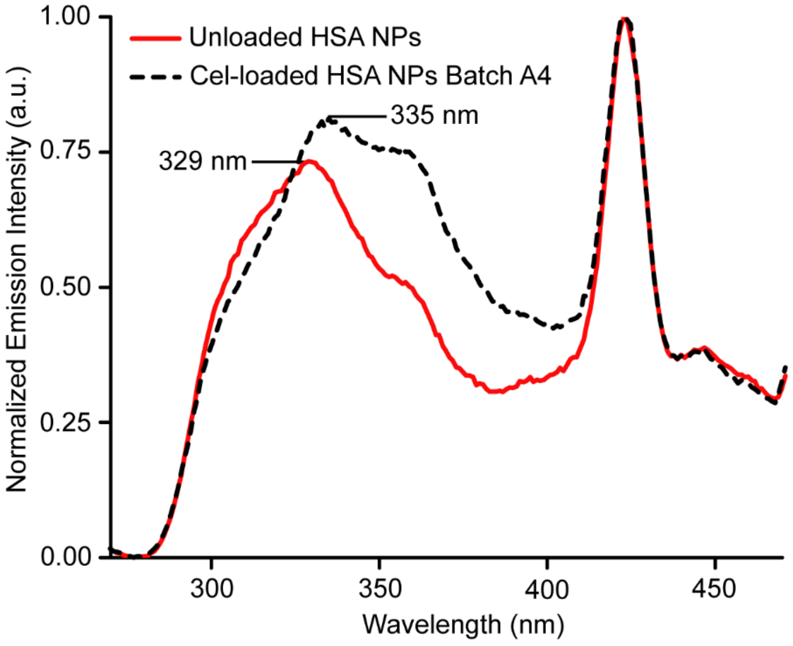

Figure S1. Fluorescence spectra (λ_{ex} = 242 nm) of aqueous suspensions of Cel-loaded HSA NPs and unloaded control HSA NPs showing the interaction of Cel with HSA in the NPs.

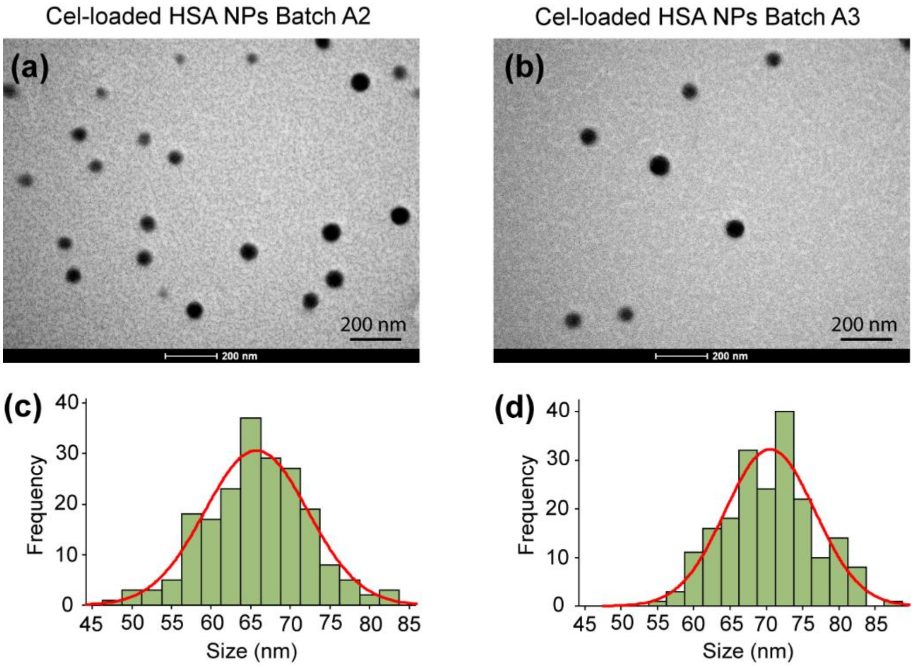

Figure S2. (a, b) TEM micrographs and (c, d) corresponding particle size distributions of two batches of Cel-loaded HSA NPs showing that the NPs meet the size criteria for penetrating in the cartilage.

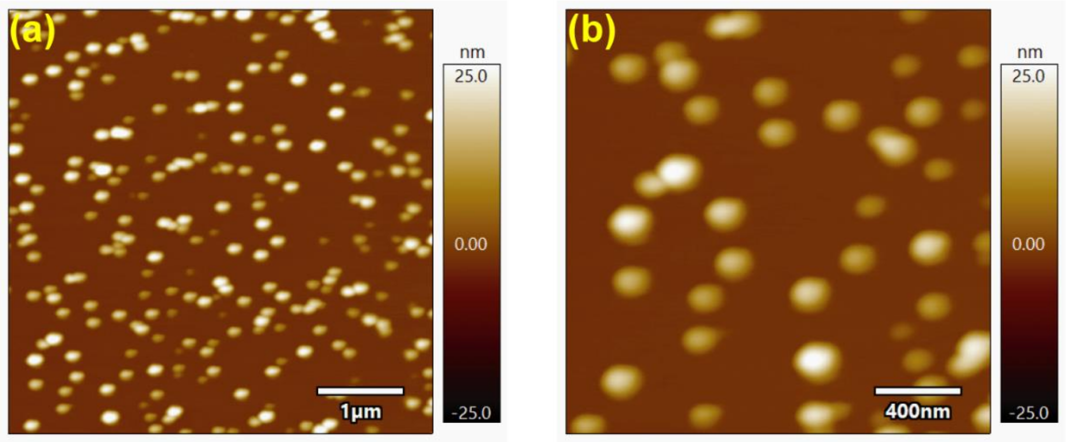

Figure S3. (a, b) AFM micrographs of Cel-loaded HSA NPs (Batch A3) showing non-agglomerated NPs.

Table S2. NP size, PDI and zeta potential of different batches of Cel-loaded HSA NPs.

	d_{TEM} (nm)		d_{hyd} (nm)		PDI by DLS		Zeta Potential (mV) by DLS	
	Synthesized using same batch of starting HSA	Synthesized using three different batches of starting HSA	Synthesized using same batch of starting HSA	Synthesized using three different batches of starting HSA	Synthesized using same batch of starting HSA	Synthesized using three different batches of starting HSA	Synthesized using same batch of starting HSA	Synthesized using three different batches of starting HSA
	60.8 (A1)	65.7* (A1, A2, A3)	100.4 (A1)	103.4* (A1, A2, A3)	0.03 (A1)	0.03* (A1, A2, A3)	-34 (A1)	-35.3* (A1, A2, A3)
	65.7 (A2)	75.4 (B1)	102.6 (A2)	124.6 (B1)	0.03 (A2)	0.03 (B1)	-37 (A2)	-36 (B1)
	70.5 (A3)	73.6 (C1)	107.1 (A3)	86.5 (C1)	0.02 (A3)	0.06 (C1)	-35 (A3)	-32 (C1)
**Average**	**65.7**	**71.6**	**103.4**	**104.8**	**0.03**	**0.04**	**-35.3**	**-34.4**
**Std dev**	**4.8**	**5.2**	**3.4**	**19.1**	**0.01**	**0.02**	**1.5**	**2.1**
**%RSD**	**7.4**	**7.2**	**3.3**	**18.2**	**21.65**	**43.30**	**4.3**	**6.2**

*Average of A1, A2 and A3.

Note - A1, A2, A3, B1 and C1 are the different batches of Cel-loaded HSA NPs.
A1, A2 and A3 were synthesized using the starting HSA Batch SLBM7779V, B1 synthesized using the starting HSA Batch SLCB2530 and C1 synthesized using the starting HSA Batch SLCF6784.

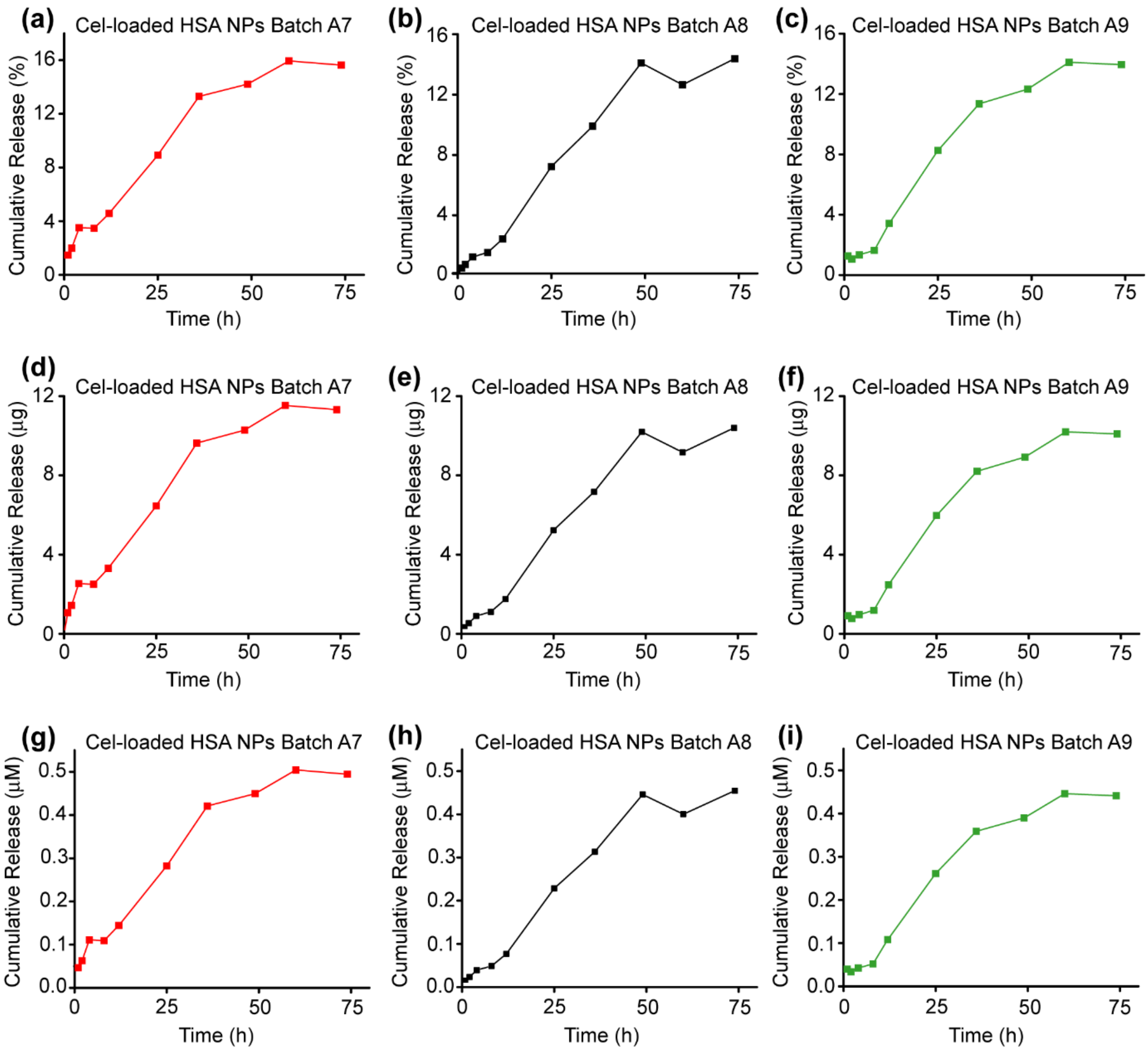

Figure S4. Cumulative release of Cel (a-c) in %, (d-f) in µg, and (g-i) in µM at 37 °C from three batches of Cel-loaded HSA NPs in PBS (pH 7.4, 60 mL) over 74 h. The release was measured by recording the emission spectra (λ_{ex} = 242 nm) of Cel in PBS and observing the intensity at 405 nm. This figure indicates that the release in PBS was slow without a burst effect and with an average half time for release of 25-30 h.

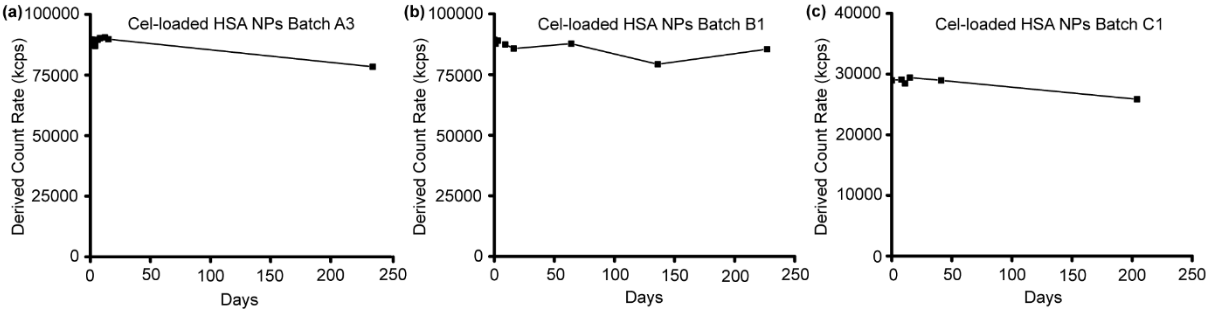

Figure S5. (a-c) Derived count rates of three batches of Cel-loaded HSA NP suspensions (maintained at 4 °C) recorded on different days. The DLS measurements were carried out at 25 °C. This demonstrates that the Cel-loaded HSA NP suspensions were stable for at least 6 months when stored at 4 °C.

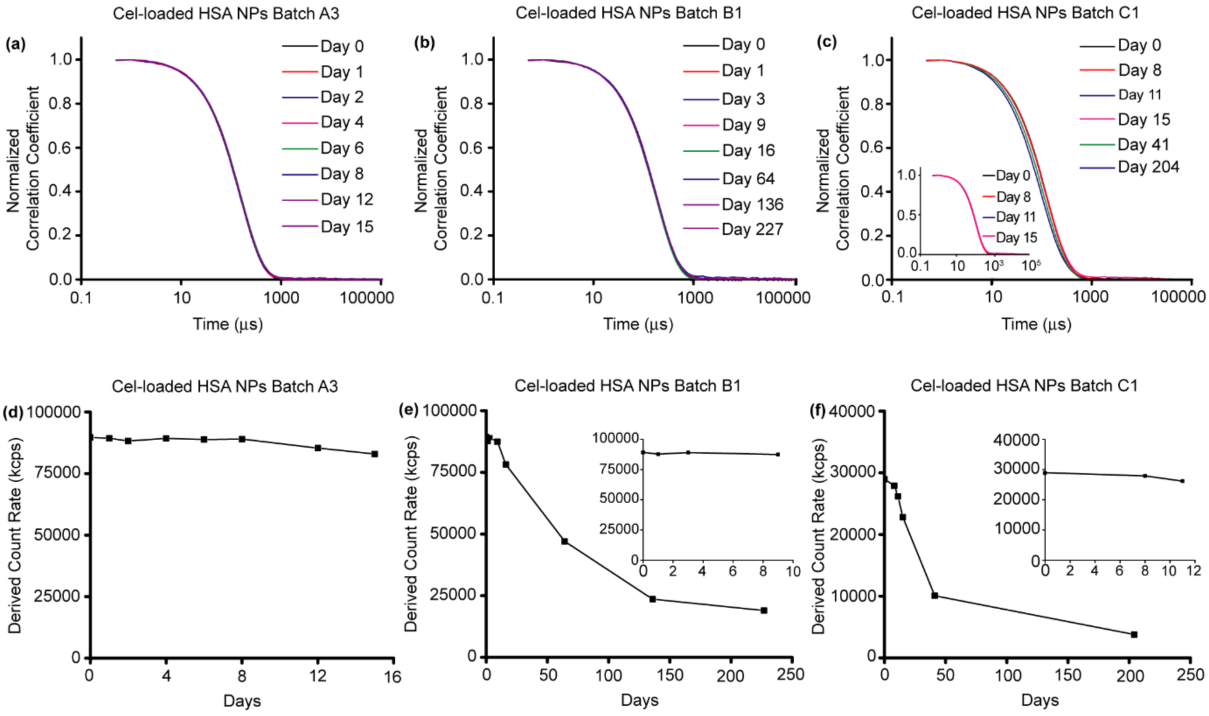

Figure S6. (a-c) DLS correlograms and (d-f) derived count rates of three batches of Cel-loaded HSA NP suspensions (maintained at 22 °C) recorded on different days. The DLS measurements were carried out at 25 °C. The inset of figure (c) shows the DLS correlograms for the Batch C1 recorded on different days till day 15, the inset of figure (e) shows the derived count rates for the Batch B1 recorded on different days till day 9, and the inset of figure (f) shows the derived count rates for the Batch C1 recorded on different days out to day 11. This figure indicates that the NPs when stored at 22 °C were stable for at least 9 days.

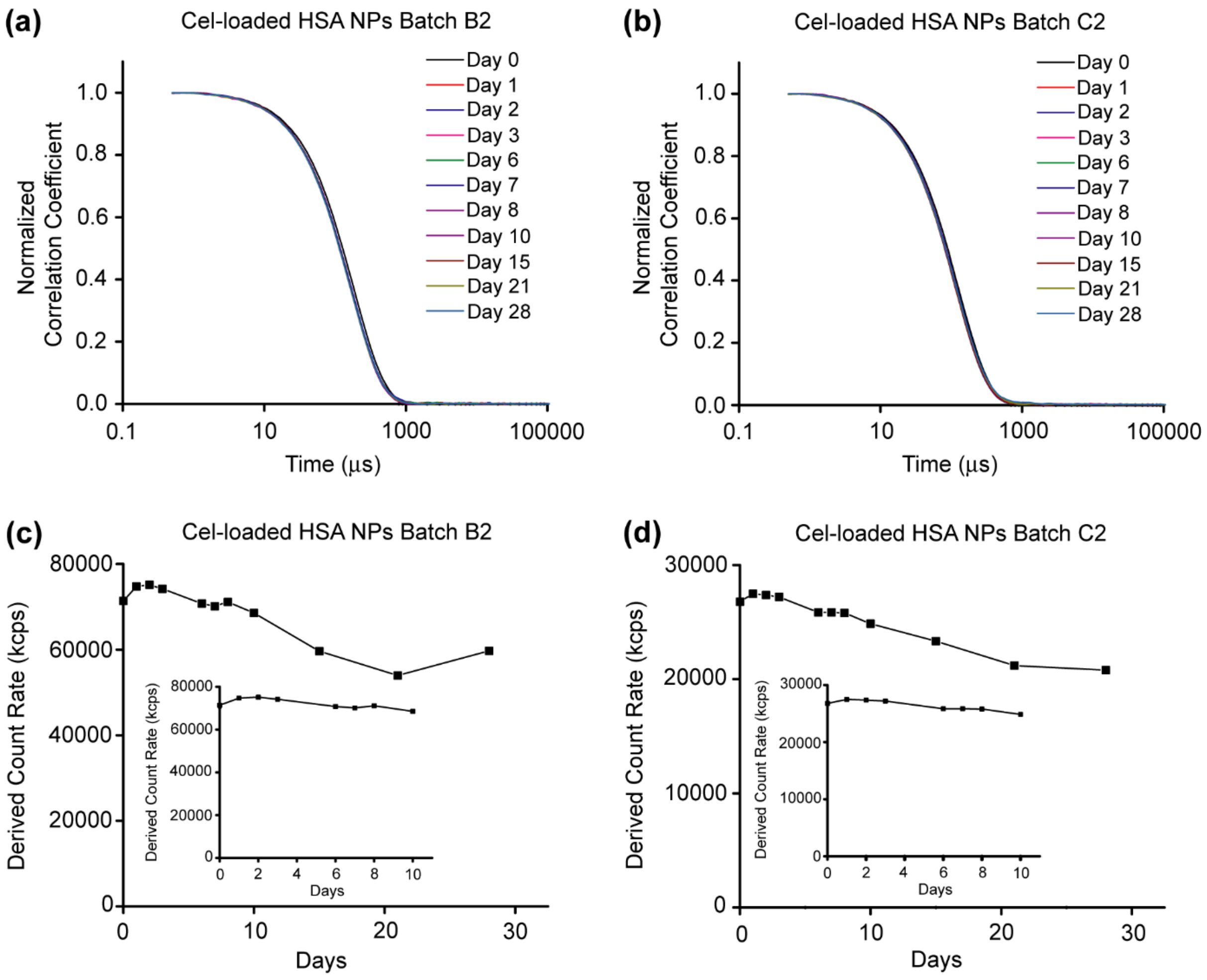

Figure S7. (a, b) DLS correlograms and (c, d) derived count rates of two batches of Cel-loaded HSA NP suspensions (maintained at 37 °C) recorded on different days. The DLS measurements were carried out at 25 °C. The insets of figures (c) and (d) show the derived count rate for the Batches B2 and C2 respectively recorded on different days till day 10. This figure indicates that the NPs were stable for at least 10 days when incubated at 37 °C.

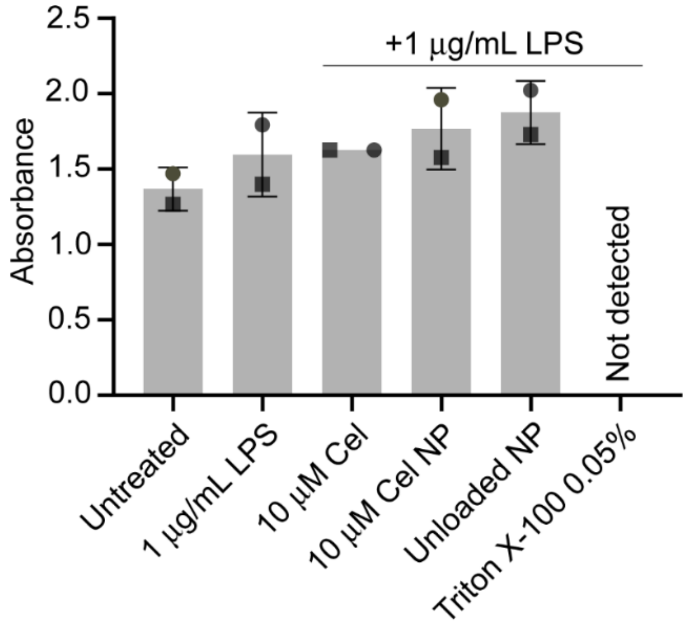

Figure S8. Effect of Cel-loaded HSA NPs on the cellular viability of LPS-stimulated THP-1 cells as assessed by MTS assay. Bars represent the mean ± SD of two biological experiments and the two data points per group represent the mean of each biological experiment. For each biological experiment, 3-4 technical replicates were obtained. For statistical analysis, a one-way ANOVA with Dunnett's *post-hoc* test was used. This figure indicates that there was no effect of Cel-loaded HSA NPs on the cellular viability of LPS-stimulated THP-1 cells.

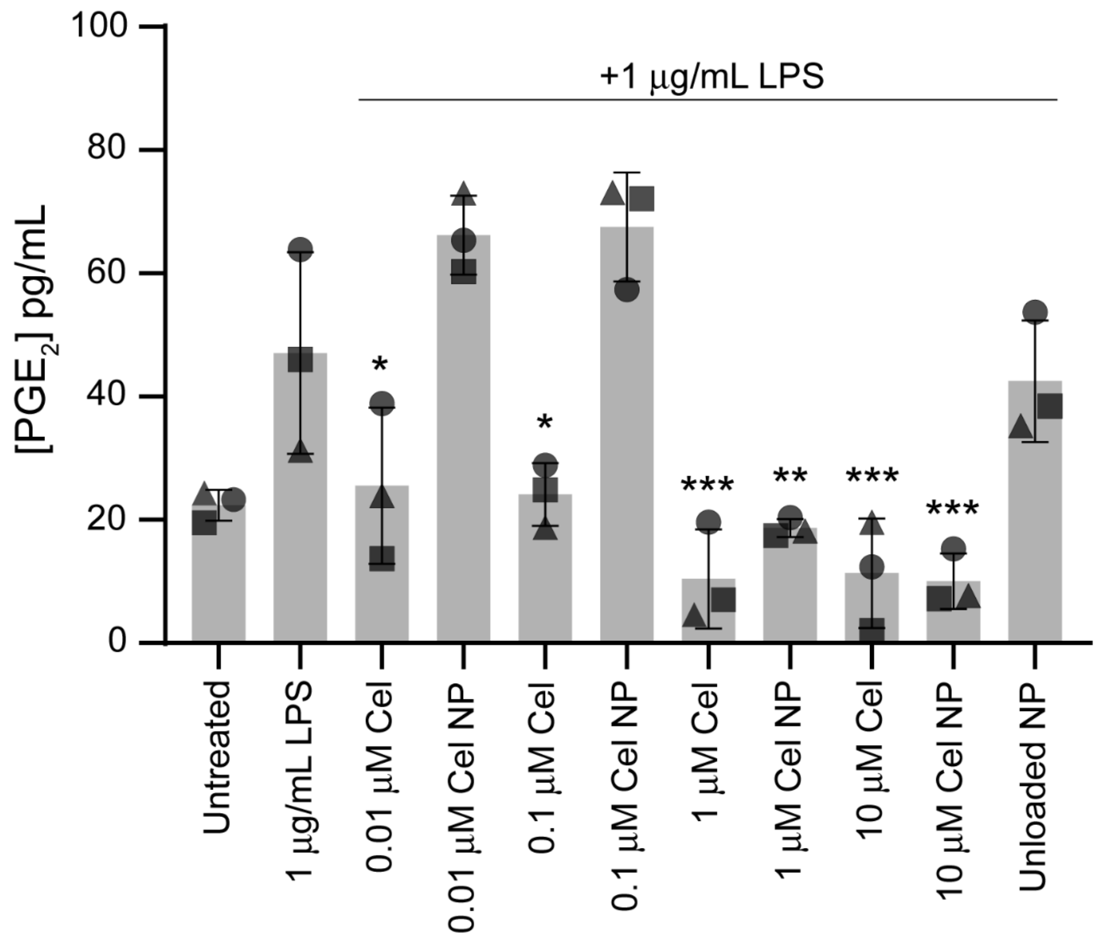

Figure S9. Reduction in PGE_2 concentrations released by LPS-stimulated THP-1 cells by both Cel and Cel-loaded HSA NPs. 0.01 μM Cel NP corresponds to the concentration of Cel-loaded HSA NP calculated to contain 0.01 μM Cel and so on. The concentration of unloaded control NP used was equivalent to the concentration of NP in 10 μM Cel NP. Bars represent the mean ± SD of three biological experiments and the three data points per group represent the mean of each biological experiment. For each biological experiment, 2-3 technical replicates were obtained. For statistical analysis, a one-way ANOVA with Dunnett's *post-hoc* test was used. *$p < 0.05$; **$p < 0.01$; ***$p < 0.001$ compared to LPS-stimulated only (second column from the left). This figure indicates that both Cel-loaded HSA NPs and Cel reduced the PGE_2 levels released from LPS-stimulated THP-1 cells.

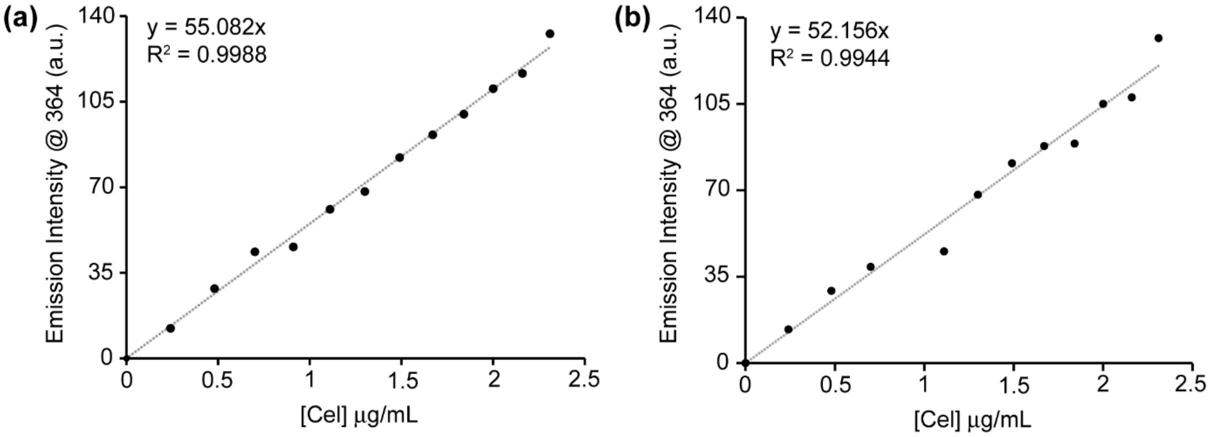

Figure S10. (a, b) Fluorescence calibration curves used for calculating the E.E. for two batches of Cel-loaded HSA NPs (Batch A5 and A6). Excitation wavelength used was 242 nm.
